# National trends in hospital length of stay for acute myocardial infarction in China

**DOI:** 10.1186/1471-2261-15-9

**Published:** 2015-01-20

**Authors:** Qian Li, Zhenqiu Lin, Frederick A Masoudi, Jing Li, Xi Li, Sonia Hernández-Díaz, Sudhakar V Nuti, Lingling Li, Qing Wang, John A Spertus, Frank B Hu, Harlan M Krumholz, Lixin Jiang

**Affiliations:** Department of Epidemiology, Harvard School of Public Health, Boston, MA USA; Epidemiology, Worldwide Safety & Regulatory, Pfizer Inc., New York, NY USA; Center for Outcomes Research and Evaluation, Yale University School of Medicine, New Haven, CT USA; Division of Cardiology, University of Colorado Anschutz Medical Campus, Aurora, CO USA; National Clinical Research Center of Cardiovascular Diseases, State Key Laboratory of Cardiovascular Disease, Fuwai Hospital, National Center for Cardiovascular Diseases, Chinese Academy of Medical Sciences and Peking Union Medical College, 167 Beilishi Road, Beijing, 100037 China; Department of Population Medicine, Harvard Medical School and Harvard Pilgrim Health Care Institute, Boston, MA USA; Saint Luke’s Mid America Heart Institute, Kansas City, MO USA; Channing Division of Network Medicine, Department of Medicine, Brigham and Women’s Hospital and Harvard Medical School, Boston, MA USA; Department of Nutrition, Harvard School of Public Health, Boston, MA USA

**Keywords:** Acute myocardial infarction, Length of stay, Variation in care

## Abstract

**Background:**

China is experiencing increasing burden of acute myocardial infarction (AMI) in the face of limited medical resources. Hospital length of stay (LOS) is an important indicator of resource utilization.

**Methods:**

We used data from the Retrospective AMI Study within the China Patient-centered Evaluative Assessment of Cardiac Events, a nationally representative sample of patients hospitalized for AMI during 2001, 2006, and 2011. Hospital-level variation in risk-standardized LOS (RS-LOS) for AMI, accounting for differences in case mix and year, was examined with two-level generalized linear mixed models. A generalized estimating equation model was used to evaluate hospital characteristics associated with LOS. Absolute differences in RS-LOS and 95% confidence intervals were reported.

**Results:**

The weighted median and mean LOS were 13 and 14.6 days, respectively, in 2001 (n = 1,901), 11 and 12.6 days in 2006 (n = 3,553), and 11 and 11.9 days in 2011 (n = 7,252). There was substantial hospital level variation in RS-LOS across the 160 hospitals, ranging from 9.2 to 18.1 days. Hospitals in the Central regions had on average 1.6 days (p = 0.02) shorter RS-LOS than those in the Eastern regions. All other hospital characteristics relating to capacity for AMI treatment were not associated with LOS.

**Conclusions:**

Despite a marked decline over the past decade, the mean LOS for AMI in China in 2011 remained long compared with international standards. Inter-hospital variation is substantial even after adjusting for case mix. Further improvement of AMI care in Chinese hospitals is critical to further shorten LOS and reduce unnecessary hospital variation.

**Electronic supplementary material:**

The online version of this article (doi:10.1186/1471-2261-15-9) contains supplementary material, which is available to authorized users.

## Background

China, like many other low- and middle-income countries, is challenged to provide care for a large and growing population with cardiovascular conditions [1]. It is estimated that 16 million people will suffer acute myocardial infarction (AMI) in 2020 and 23 million in 2030 in China [2]. However, the country has limited medical structural resources to dedicate to the care of this increasingly common condition. The availability of hospital beds is limited; but, paradoxically, studies suggest that hospital length of stay (LOS) is longer in China compared with most other countries [3–6], which further strains resource availability. Prolonged hospitalization can expose patients to harm, including risks for hospital-acquired infections, deep vein thrombosis, pulmonary embolism, and medical errors [7, 8]. Moreover, days in hospital that do not contribute to meaningful improvements in patients’ conditions represent wasteful health care spending [8–10].

AMI is a particularly suitable condition to study hospital LOS in China. It is a common condition for which people seek acute care in a wide spectrum of hospitals. Furthermore, standardized care strategies for AMI are relatively well-established, and consistent by national [11, 12] and international [13–16] guidelines. There is also an extensive body of literature demonstrating that shorter LOS for patients with AMI is not associated with worse post-discharge outcomes, such as readmissions or mortality [17–20]. Some studies have even shown that discharge within 72 hours for low-risk and uncomplicated patients with AMI can be safe [21–24]. To this end, several risk-stratification strategies have been suggested to triage patients into different levels of readiness for discharge [8].

Despite the importance of this issue, relatively little is known about the patterns of hospital LOS for patients with AMI across China, with evidence mainly from single or very selective tertiary hospitals in urban settings [5, 6]. Accordingly, we examined LOS for AMI in a nationally representative sample of patients hospitalized for AMI during 2001, 2006, and 2011, which is derived from the China Patient-centered Evaluative Assessment of Cardiac Events (PEACE)-Retrospective AMI Study. We specifically sought to examine the variation in LOS across hospitals and over time in China and to identify hospital characteristics that are associated with shorter LOS, employing methods specifically developed for profiling hospitals.

## Methods

### Design overview of the China PEACE-retrospective AMI study

The design of the China PEACE-Retrospective AMI Study has been published previously [25, 26]. In brief, a nationally representative sample of AMI hospitalizations was obtained following a two-stage sampling design: First, we identified hospitals using a simple random sampling procedure within each of the 5 study strata: Eastern-rural, Central-rural, Western-rural, Eastern-urban, and Central/Western-urban regions, since hospital volumes and clinical capacities differ between urban and rural areas as well as among the three official economic-geographic regions (Eastern, Central, and Western) of Mainland China. We considered Central and Western urban regions together given their similar per capita income and health services capacity. In the 3 rural strata, the sampling framework consisted of the central hospital in each of the predefined rural regions (2010 central hospitals in 2010 rural regions). In the 2 urban strata, the sampling framework consisted of the highest-level hospitals in each of the predefined urban regions (833 hospitals in 287 urban regions). Since the majority of hospitals in China are publicly owned and administered, hospital closure is rare. We selected representative hospitals from 2011 to reflect current practices and traced this cohort of hospitals backward to 2006 and 2001 to describe temporal trends. Second, we drew hospitalizations from the selected hospitals based on the local hospital database for patients with a definite discharge diagnosis of AMI in each year using random sampling procedures. Patients with AMI were identified using International Classification of Diseases - Clinical Modification codes, including versions 9 (410.xx) and 10 (I21.xx), when available or through principal discharge diagnosis terms. Information on the patient characteristics, in-hospital treatments, and outcomes were extracted from the medical records. Hospital characteristics were derived from a standardized survey to all the selected hospitals, as well as information from medical records.

The central ethics committee at the China National Center for Cardiovascular Diseases approved the China PEACE-Retrospective AMI Study. All collaborating hospitals accepted the central ethics approval except for five hospitals, which obtained local approval. The Chinese government, who provided financial support for the study, had no role in the design or conduct of the study; in the collection, management, analysis, and interpretation of the data; or in the preparation or approval of the manuscript. The study is registered at www.clinicaltrials.gov (NCT01624883).

### Study sample

**Figure 1:**
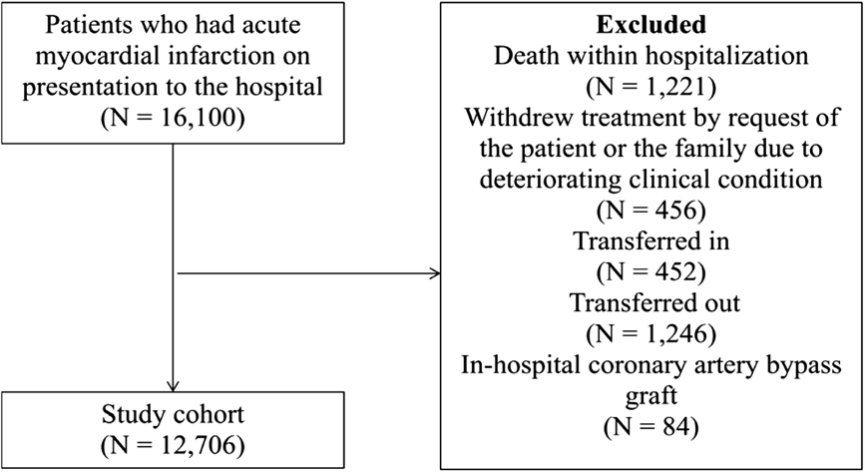
**Flow diagram of study population.**

Our study sample included 16,100 patients who had AMI on presentation to the hospital. We excluded those who died within hospital, who withdrew treatment by request of the patient or the family due to deteriorating clinical condition, who transferred into or out of the hospital, or who had a coronary artery bypass graft (CABG) during hospitalization (Figure 1). In a secondary analysis, we considered only the subgroup of patients without major complications during hospitalization, for whom an extended LOS maybe particularly unnecessary. Uncomplicated patients were defined as those without recurrent myocardial infarction or angina, cardiogenic shock, cardiac arrest, new-onset heart failure, atrial fibrillation, ventricular tachycardia or fibrillation, stroke, bleeding, acute renal failure, or infection during hospitalization.

### Covariates

#### Patient-level characteristics

Data elements were identified from the front page of the medical record (i.e. patient’s sociodemographic characteristics and a summary of major events during hospitalization), admission records (including conditions at presentation, history of disease, personal lifestyle and reproductive history, physical examination, and auxiliary examination), daily records (events during hospitalization), in-hospital procedure reports (e.g. device placement, coronary angiography, percutaneous coronary intervention (PCI), and CABG), diagnostic examination reports (e.g. imaging, electrocardiography, and lab tests), long-term and short-term physician orders, and discharge records. Variables were classified into categories, including sociodemographic and medical characteristics; diagnostic tests, medications, and procedures received during hospitalization; and in-hospital outcomes.

#### Hospital-level characteristics

Hospital characteristics included geographic region (Eastern/Central/Western), hospital level (tertiary/secondary), teaching status, annual AMI patient volume, availability of an independent cardiology department, cardiac catheterization, and the capacity to conduct biomarkers testing (creatine kinase, creatine kinase MB fraction, and troponin).

### Outcome

LOS in days was primarily determined by the admission and discharge dates documented on the front page of the medical records, and complemented by other sections of the record when either date was missing, uninterpretable, or mis-documented on the front page.

### Statistical analysis

Hospitals were divided into tertiles based on the median LOS across all years. To assess statistical differences and take into account the clustering of patients within hospitals, patient characteristics were compared across the tertiles of hospitals using the Chi-square test or ANOVA for clustered data. We also examined the trends in LOS over the study period among all patients and in uncomplicated patients only. Mann-Kendall trend test was used based on three time points (2001, 2006, and 2011). When deriving the patient-level LOS statistics, the weight for each patient was the inversed sampling fraction of patients from the hospital multiplied by the inverse sampling fraction of hospitals from the geographic region for each study year.

Our subsequent analytic cohort further excluded patients who had LOS lower than the 1st percentile value (i.e. who were discharged on the same day or overnight) or greater than the 99th percentile value (>42 days). LOS was considered a log-normal distribution. In the overall study sample, two-level generalized linear mixed model with patients nested within hospitals was used to determine the risk-standardized LOS (RS-LOS). The “risk profile” consisted of factors that may be associated with the hospitalization LOS, including patient’s sociodemographics, cardiac and noncardiac comorbidities, indicators of the severity of AMI, and year. RS-LOS for each patient was defined as the ratio of predicted to expected LOS, multiplied by the average LOS of the cohort. The expected LOS for each patient was estimated by applying the estimated regression coefficients to the characteristics of the patient and adding the average of the 160 hospital-specific intercepts. The predicted LOS of each patient was calculated with similar methods but using individual hospital-specific intercepts instead of the average value. Therefore, although RS-LOS was technically calculated for each patient based on the model, it was really a hospital-specific indicator (i.e. patients within the same hospital had identical RS-LOS).

Furthermore, we assessed inter-hospital variation in RS-LOS across the study years, among all hospitals as well as stratified by secondary and tertiary hospital levels. We did not adjust for patient in-hospital treatments and complications as they could act as mediators, and adjustment for these factors may result in underestimates of variation.

Lastly, we examined the association of year-specific hospital RS-LOS with the selected hospital characteristics, some of which might change over the years for the same hospital. Year-specific hospital RS-LOS was derived similarly as described above, except it was estimated separately in patients from each of three study years. We fit a generalized estimating equation (GEE) linear model, weighted by hospitals’ AMI patient volume for each study year in the study sample to account for the uncertainty in the estimates of RS-LOS, and took into account the correlations of different year-specific observations for the same hospital. Absolute differences in RS-LOS and 95% confidence intervals (CIs) were reported for each hospital characteristic, adjusting for temporal (i.e. year) effect.

All comparisons were 2-sided, with a p-value less than 0.05 considered statistically significant. Statistical analysis was performed using the SAS software (version 9.3, SAS Institute, Cary, NC).

## Results

### Characteristics of patients in tertiles of hospitals with regard to crude median LOS

The median and interquartile range (IQR) of LOS was 13 (8–19) days in 2001, 11 (7–16) days in 2006, and 11 (7–15) days in 2011 (Table 1); the rankings of the average LOS of the three tertiles of hospitals were consistent throughout the three study years. Compared with hospitals in the low tertile of median LOS, high-tertile hospitals contributed more patients to our study sample in 2001 (16.2% vs. 10.4%) and fewer in 2011 (55.4% vs. 61.1%). With respect to patient characteristics by tertiles of hospitals, high-tertile hospitals had a greater proportion of current smokers (38.4%) and a lower proportion of patients with chronic renal disease (16.7%); middle-tertile hospitals had a greater proportion of patients with a history of MI (12.5%); and low-tertile hospitals had fewer patients with estimated glomerular filtration rate > 90 ml/(min*1.73 m^2^) (26.0%). Other patient characteristics were not statistically different across the three tertiles of hospitals. Of note, with regard to the distribution of outliers in LOS, low-tertile hospitals had more patients who had LOS of 0 or 1 day (4.0%), while high-tertile hospitals had more patients who had LOS of >42 days (1.4%).

**Table 1:** **Characteristics of patients in tertiles of hospitals with regard to median length of stay**

	All hospitals (n = 12706)	High-tertile hospitals (n = 4784)	Middle-tertile hospitals (n = 5105)	Low-tertile hospitals (n = 2817)	P-value
Length of stay, median (IQR)					
2001	13 (8–19)	14 (9–21)	14 (9–18)	11 (7–16)	0.004
2006	11 (7–16)	13 (9–19)	11 (7–15)	8 (5–12)	<.0001
2011	11 (7–15)	14 (9–18)	11 (8–14)	9 (6–12)	<.0001
Year of admission					0.01
2001	1901 (15.0)	773 (16.2)	834 (16.3)	294 (10.4)	
2006	3553 (28.0)	1361 (28.5)	1391 (27.3)	801 (28.4)	
2011	7252 (57.1)	2650 (55.4)	2880 (56.4)	1722 (61.1)	
**Socio-demographics**					
Age, years					
Mean ± std	64.4 ± 12.5	63.8 ± 12.4	64.5 ± 12.7	65.1 ± 12.1	0.2
<55	2905 (22.9)	1176 (24.6)	1172 (23.0)	557 (19.8)	0.07
55-64	3073 (24.2)	1125 (23.5)	1237 (24.2)	711 (25.2)	
65-74	3779 (29.7)	1455 (30.4)	1460 (28.6)	864 (30.7)	
≥75	2949 (23.2)	1028 (21.5)	1236 (24.2)	685 (24.3)	
Female	3779 (29.7)	1385 (29.0)	1574 (30.8)	820 (29.1)	0.4
Place of residence					0.1
Urban	3489 (27.5)	1525 (31.9)	1316 (25.8)	648 (23.0)	
Rural	8266 (65.1)	3087 (64.5)	3166 (62.0)	2013 (71.5)	
Unrecorded	951 (7.5)	172 (3.6)	623 (12.2)	156 (5.5)	
**Cardiac risk factors**					
Current smoking	4406 (34.7)	1836 (38.4)	1659 (32.5)	911 (32.3)	0.03
Hypertension	5743 (45.2)	2252 (47.1)	2323 (45.5)	1168 (41.5)	0.3
Diabetes	2095 (16.5)	805 (16.8)	914 (17.9)	376 (13.4)	0.1
**Medical histories**					
Myocardial infarction	1369 (10.8)	466 (9.7)	639 (12.5)	264 (9.4)	0.008
Percutaneous coronary intervention	248 (2.0)	95 (2.0)	113 (2.2)	40 (1.4)	0.4
CABG	45 (0.4)	21 (0.4)	19 (0.4)	5 (0.2)	0.4
Stroke	1377 (10.8)	517 (10.8)	568 (11.1)	292 (10.4)	0.9
Chronic renal disease	2527 (19.9)	799 (16.7)	1076 (21.1)	652 (23.2)	0.02
Cancer	72 (0.6)	14 (0.5)	21 (0.4)	37 (0.8)	0.1
**Presentation features**					
Symptom onset to admission					0.5
≤6 hours	4838 (38.1)	1891 (39.5)	1925 (37.7)	1022 (36.3)	
6-12 hours	1290 (10.2)	477 (10.0)	525 (10.3)	288 (10.2)	
12-24 hours	1635 (12.9)	635 (13.3)	626 (12.3)	374 (13.3)	
>24 hours	4943 (38.9)	1781 (37.2)	2029 (39.8)	1133 (40.2)	
STEMI	10888 (85.7)	4190 (87.6)	4293 (84.1)	2405 (85.4)	0.07
Chest pain	11746 (92.4)	4421 (92.4)	4719 (92.4)	2606 (92.5)	0.9
Cardiogenic shock	475 (3.7)	190 (4.0)	175 (3.4)	110 (3.9)	0.6
Cardiac arrest	105 (0.8)	48 (1.0)	39 (0.8)	18 (0.6)	0.3
Pneumonia	1188 (9.4)	416 (8.7)	486 (9.5)	286 (10.2)	0.7
Exacerbated COPD	203 (1.6)	73 (1.5)	76 (1.5)	54 (1.9)	0.6
Acute stroke	114 (0.9)	41 (0.9)	43 (0.8)	30 (1.1)	0.6
eGFR, ml/min/1.73 m^2^					
Mean ± std	84.6 ± 38.6	88.7 ± 37.4	82.9 ± 37.5	80.5 ± 42.0	0.008
>90	4210 (33.1)	1848 (38.6)	1630 (31.9)	732 (26.0)	0.0006
60-90	4158 (32.7)	1515 (31.7)	1792 (35.1)	851 (30.2)	
<60	2389 (18.8)	759 (15.9)	1017 (19.9)	613 (21.8)	
Unknown	1949 (15.3)	662 (13.8)	666 (13.1)	621 (22.0)	
Systolic blood pressure >180 mmHg or diastolic blood pressure >110 mmHg	671 (5.3)	227 (4.7)	297 (5.8)	147 (5.2)	0.5
Heart rate > 100 beats/min	1379 (10.9)	493 (10.3)	572 (11.2)	314 (11.2)	0.5
**Outliers of outcome**					
<1st percentile value (i.e. <2 days)	316 (2.5)	102 (2.1)	101 (2.0)	113 (4.0)	0.001
>99th percentile value (i.e. >42 days)	121 (1.0)	69 (1.4)	44 (0.9)	8 (0.3)	< .0001

IQR: interquartile range; std: standard deviation; CABG: coronary artery bypass graft; STEMI: ST-segment elevation myocardial infarction; COPD: chronic obstructive pulmonary disease; eGFR: estimated glomerular filtration rate.

Numbers in the second to the fifth columns represent count (percentage), unless otherwise specified.

As Table 2 shows, 8.2% of the patients in high-tertile hospitals received no biomarker testing in contrast to 17.0% in low-tertile hospitals. Percentages of medication use, reperfusion therapies, and overall cardiac procedures during the hospitalization were similar between patients in hospitals from the three tertiles. In-hospital outcomes were also similar (Table 3), except for in-hospital infection – 12.6% of the patients had infections acquired during hospitalization in high-tertile hospitals, whereas the proportions were 10.8% and 8.7% for middle- and low-tertile hospitals, respectively.

**Table 2:** **In-hospital diagnostic tests, treatments, and procedures received by patients in tertiles of hospitals with regard to median length of stay**

	All hospitals (n = 12706)	High-tertile hospitals (n = 4784)	Middle-tertile hospitals (n = 5105)	Low-tertile hospitals (n = 2817)	P-value
Diagnostic tests					
Echocardiogram	6994 (55.0)	2765 (57.8)	2803 (54.9)	1426 (50.6)	0.6
Ejection fraction					
Available	6436 (50.7)	2497 (52.2)	2586 (50.7)	1353 (48.0)	0.8
Mean ± std	54.0 ± 11.8	54.6 ± 11.8	53.9 ± 11.6	53.3 ± 12.3	0.5
Biomarkers					0.04
None	1338 (10.5)	393 (8.2)	466 (9.1)	479 (17.0)	
CK only	855 (6.7)	263 (5.5)	399 (7.8)	193 (6.9)	
CK-MB only	4685 (36.9)	2094 (43.8)	1650 (32.3)	941 (33.4)	
Troponin only	395 (3.1)	112 (2.3)	231 (4.5)	52 (1.9)	
CK-MB + Troponin	5433 (42.8)	1922 (40.2)	2359 (46.2)	1152 (40.9)	
Medications within 24 hours of admission					
Aspirin	11209 (88.2)	4191 (87.6)	4591 (89.9)	2427 (86.2)	0.2
Clopidogrel	7418 (58.4)	2739 (57.3)	3104 (60.8)	1575 (55.9)	0.7
GP IIb/IIIa inhibitors	877 (6.9)	306 (6.4)	392 (7.7)	179 (6.4)	0.8
Unfractionated heparin	9753 (76.8)	3740 (78.2)	3840 (75.2)	2173 (77.1)	0.6
Low-molecular-weight heparin	6953 (54.7)	2692 (56.3)	2742 (53.7)	1519 (53.9)	0.8
Beta-blockers	6328 (49.8)	2346 (49.0)	2657 (52.1)	1325 (47.0)	0.4
Nitrates	10677 (84.0)	3989 (83.4)	4325 (84.7)	2363 (83.9)	0.9
Traditional Chinese medicines	7251 (57.1)	2736 (57.2)	2680 (52.5)	1835 (65.1)	0.3
Medications during the hospitalization					
Calcium channel blockers	2206 (17.4)	838 (17.5)	893 (17.5)	475 (16.9)	0.9
ACEIs or ARBs	8345 (65.7)	3226 (67.4)	3311 (64.9)	1808 (64.2)	0.7
Any Statins	9833 (77.4)	3683 (77.0)	4058 (79.5)	2092 (74.3)	0.5
Traditional Chinese medicines	8605 (67.7)	3314 (69.3)	3210 (62.9)	2081 (73.9)	0.4
Reperfusion therapies among STEMI					
Primary PCI	1307 (12.0)	506 (12.1)	606 (14.1)	195 (8.1)	0.5
Fibrinolytic therapy	2637 (24.2)	1108 (26.4)	976 (22.7)	553 (23.0)	0.4
Cardiac procedures					
Catheterization	3483 (27.4)	1357 (28.4)	1457 (28.5)	669 (23.8)	0.8
PCI	3013 (23.7)	1165 (24.4)	1284 (25.2)	564 (20.0)	0.7

CK: creatine kinase; CK-MB: creatine kinase MB fraction; ACEI: angiotensin converting enzyme inhibitor; ARB: angiotensin receptor blocker; STEMI: ST-segment elevation myocardial infarction; PCI: Percutaneous coronary intervention.

Numbers in the second to the fifth columns represent count (percentage), unless otherwise specified.

**Table 3:** **In-hospital outcomes of patients in tertiles of hospitals with regard to median length of stay**

	All hospitals (n = 12706)	High-tertile hospitals (n = 4784)	Middle-tertile hospitals (n = 5105)	Low-tertile hospitals (n = 2817)	P-value
Recurrent myocardial infarction	67 (0.5)	25 (0.5)	25 (0.5)	17 (0.6)	0.8
Cardiac arrest	124 (1.0)	55 (1.2)	47 (0.9)	22 (0.8)	0.3
Cardiogenic shock	154 (1.2)	63 (1.3)	60 (1.2)	31 (1.1)	0.7
New-onset heart failure	1656 (13.0)	612 (12.8)	688 (13.5)	356 (12.6)	0.9
Recurrent angina	2120 (16.7)	883 (18.5)	702 (13.8)	535 (19.0)	0.08
Atrial fibrillation	310 (2.4)	127 (2.7)	104 (2.0)	79 (2.8)	0.2
Stroke	63 (0.5)	29 (0.6)	22 (0.4)	12 (0.4)	0.4
Bleeding	769 (6.1)	321 (6.7)	314 (6.2)	134 (4.8)	0.2
Acute renal failure	66 (0.5)	26 (0.5)	31 (0.6)	9 (0.3)	0.4
Ventricular tachycardia or fibrillation	426 (3.4)	175 (3.7)	163 (3.2)	88 (3.1)	0.7
Infection	1397 (11.0)	601 (12.6)	551 (10.8)	245 (8.7)	0.04

Numbers in the second to the fifth columns represent count (percentage), unless otherwise specified.

### Year trend of Hospital LOS for AMI

**Figure 2:**
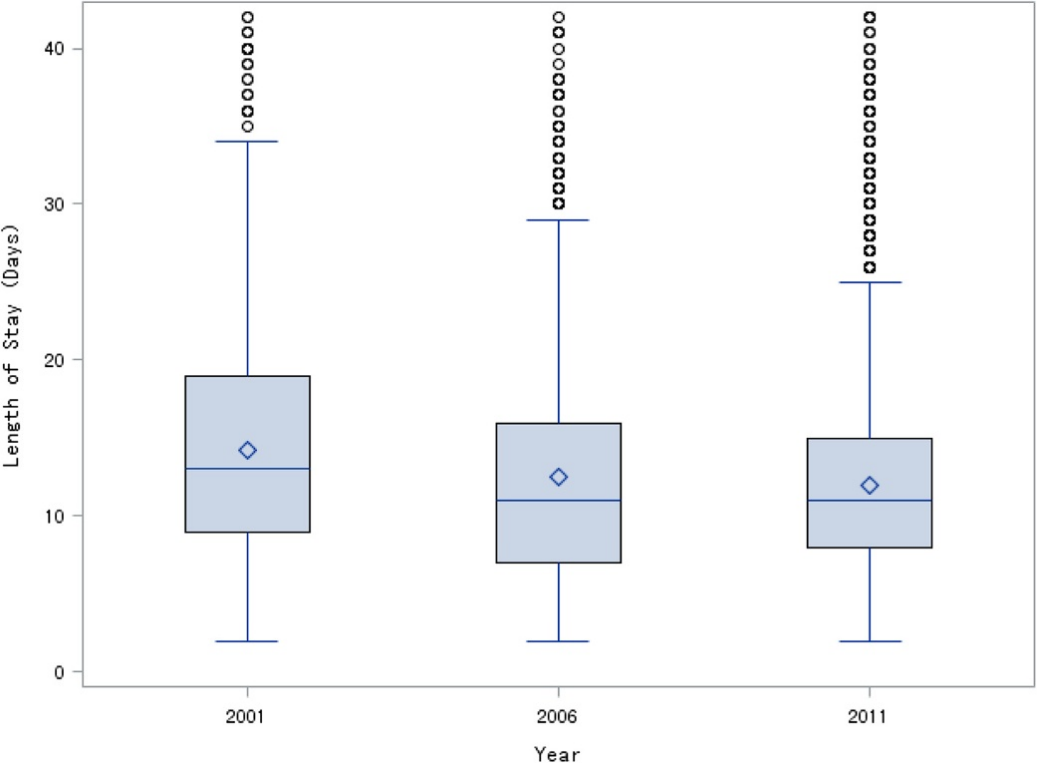
**Year-trend Whisker plot of length of stay.** Diamond inside the box: mean; line inside the box: median; bottom and top edges of the box: interquartile range (IQR); bottom and top edges of the whiskers: 1.5*IQR; points beyond the whiskers: outliers.

Compared with 2001, LOS for patients with AMI decreased in 2006 and 2011 (p for trend <0.001) (Figure 2). After weighting, among the 1,901 patients from 2001 the mean LOS was 14.6 days. The corresponding LOS for 2006 (n = 3,553) and 2011 (n = 7,252) were 12.6 and 11.9 days, respectively. Among the 8,049 uncomplicated patients, the weighted mean LOS was 13.4 days for the 1,196 patients from 2001, 11.2 days for the 2,243 patients from 2006, and 10.8 days for the 4,610 patients from 2011.

In the multivariable risk-standardization model, after adjusting for other patient characteristics, study year was significantly associated with LOS for AMI. Compared with a patient with the same underlying risk in 2011, a patient in 2001 had a 25% longer LOS (ratio, 1.25; 95% CI, 1.22-1.29) and a patient in 2006 had an 8% longer LOS (ratio: 1.08; 95% CI, 1.06-1.10). Further information about the model coefficients and the residual plot of the risk-standardization model are provided in the Additional file 1.

### Variations in risk-standardized LOS across Hospitals

**Figure 3:**
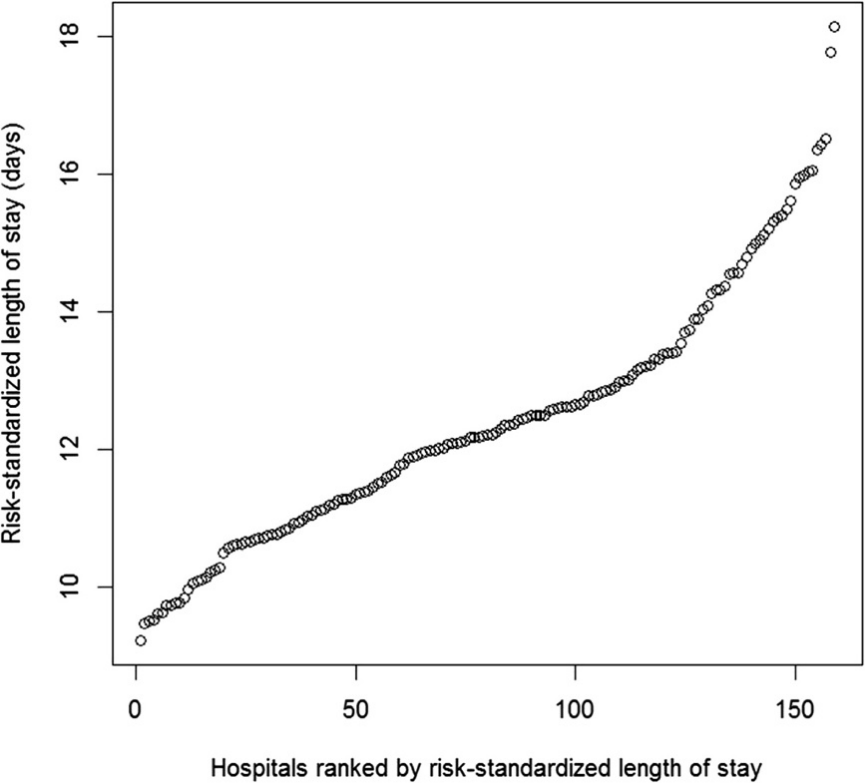
**Risk-standardized length of stay across all hospitals.**

Overall mean hospital RS-LOS was 12.5 (s.d. 1.8 days). Among all 160 hospitals, there was a substantial variation in hospital RS-LOS, ranging from 9.2 to 18.1 days (Figure 3). The patterns of variations were similar between secondary and tertiary hospitals. Among 95 secondary hospitals, the mean hospital RS-LOS was 12.0 days, with a s.d. of 1.7 days and a range of 9.2 to 18.1 days (see Additional file 2 for the graphic pattern of variation). Among 65 tertiary hospitals, the mean hospital RS-LOS was 12.8 days, with a s.d. of 1.7 days and a range of 9.5 to 17.8 days (see Additional file 3 for the graphic pattern of variation).

### Hospital characteristics associated with risk-standardized LOS

After adjusting the standard errors of estimates using GEE, geographic region was the only factor independently significantly associated with hospital RS-LOS (Table 4). Compared with hospitals in the Eastern region, Central-region hospitals on average had 1.6 days (p = 0.02) shorter RS-LOS. All other hospital characteristics relating to capacity for AMI treatment were not significantly associated with LOS.

**Table 4:** **Hospital characteristics associated with year-specific risk-standardized length of stay**

Hospital characteristics	N (%)			Difference in risk-standardized length of stay and 95%confidence intervals (in days)	P-value
	2001	2006	2011		
	(n = 130)	(n = 152)	(n = 158)		
Geographic region					
Eastern	55 (42.3)	60 (39.5)	63 (39.9)	0 (ref)	
Central	41 (31.5)	48 (31.6)	48 (30.4)	−1.6 (−2.9, −0.2)	0.02
Western	34 (26.2)	44 (29.0)	47 (29.8)	0.3 (−1.1, 1.6)	0.7
Tertiary (vs. secondary)	56 (43.1)	64 (42.1)	65 (41.1)	0.5 (−0.7, 1.7)	0.4
Affiliated/teaching status					
Neither affiliated nor teaching	37 (28.5)	42 (27.6)	42 (26.6)	0 (ref)	
Teaching but not affiliated	48 (36.9)	53 (34.9)	54 (34.2)	0.2 (−1.0, 1.3)	0.8
Both teaching and affiliated	45 (34.6)	57 (37.5)	62 (39.2)	−0.4 (−2.1, 1.2)	0.6
Annual AMI patients volume					
≤10	58 (44.6)	39 (25.7)	17 (10.8)	0 (ref)	
11-30	37 (28.5)	43 (28.3)	29 (18.4)	0.1 (−0.9, 1.2)	0.8
31-80	25 (19.2)	35 (23.0)	52 (32.9)	0.6 (−0.7, 1.8)	0.4
>80	10 (7.7)	35 (23.0)	60 (38.0)	0.8 (−0.6, 2.2)	0.3
Cardiac catheterization (vs. not)	33 (25.4)	58 (38.2)	74 (46.8)	0 (−0.5, 0.5)	0.9
Independent cardiology department (vs. not)	28 (21.5)	58 (38.2)	78 (49.4)	−0.3 (−0.7, 0.2)	0.2
Capacity to test biomarkers					
None	24 (18.5)	11 (7.2)	2 (1.3)	0 (ref)	
CK only	29 (22.3)	9 (5.9)	2 (1.3)	0.1 (−1.1, 1.3)	0.9
CK-MB	44 (33.9)	44 (29.0)	27 (17.1)	0.2 (−0.8, 1.2)	0.7
Troponin	33 (25.4)	88 (57.9)	127 (80.4)	0.4 (−0.6, 1.5)	0.4

CK: creatine kinase; CK-MB: creatine kinase MB fraction.

## Discussion

In this study, we found that the average LOS for patients with AMI in China decreased by 3 days between 2001 and 2011. However, it remained considerably long, with a mean of 12 days in 2011. Even among patients without major complications, the mean LOS was 11 days. Variations in LOS across both secondary and tertiary hospitals were significant and persisted after adjusting for case mix. Regional differences were also observed, as hospitals from the Central region had about a day and a half shorter LOS for patients with AMI compared with Eastern region hospitals. Finally, hospital capacity for AMI treatment was not associated with LOS.

The LOS for AMI in China is longer than that in clinical practice of Western countries. Existing literature suggests that the average LOS for AMI patients ranges from three to eight days for most developed countries during our study period [3, 17, 19, 27, 28], with an exception of Japan, where LOS is as long as 17–20 days [29]. Contemporary trends and hospital variation in LOS for patients with AMI in China have not been previously reported. Our study shows that LOS for uncomplicated patients with AMI in China is still considerably long, suggesting that the duration of the hospital stay does not reflect patient needs. Smith et al. [23] reported that many doctors across different countries are still conservative in terms of discharge, especially facing patients with AMI who are usually elderly and with many comorbidities, which is likely the case in China as well. In addition, the post-hospitalization care for AMI patients, such as cardiac rehabilitation and clinician’s follow-up check-ups, were not as common in China as in the Western countries [30–32]. Considering that timely admission after symptom onset is still problematic for patients with AMI [26], and patients post-AMI likewise, the potential consequence of developing complications outside hospital might be even more deleterious for patients with AMI in China. Moreover, Chinese doctors tend to be conservative to avoid potential challenges, legal action, or even threats from patients or their families, who might get angry if they are sent home and have a complication [33, 34].

The structure of healthcare financing may also partially explain why LOS is longer in China than in other countries [35]. For example, the U.S. Medicare prospective payment system and diagnosis related group (DRG)-based payment system for hospitalizations gives predetermined reimbursements to hospitals independently of LOS, providing financial incentives for early discharge. China, on the other hand, largely uses a fee-for-service mechanism [36], which may incentivize those providers or hospitals where hospital beds outnumber patient demand to prolong patient’s hospitalization and generate more revenue. In addition, in many health insurance schemes in China, the amount of reimbursement to patients is higher for in-patient service than in the outpatient setting [37, 38]. Therefore, some patients may opt to stay in hospital longer in order to have other comorbid conditions checked or treated, and have more medications prescribed during hospitalization or at discharge.

The declining trend of LOS for AMI in our study is consistent with what has been observed in many other countries over time [18, 19, 39]. There are various reasons for this observation. For example, in the U.S., the DRG-based payment system provides major incentives, in addition to the increasing adoption of hospitalist programs that help to decrease LOS [40]. In China, it could be due to increases in biomarker testing that may have led to faster diagnosis and treatment, increasing use of some guideline-recommended therapies as we saw in the China PEACE-Retrospective AMI Study data [26], or system and organizational strategy improvements [41].

We found substantial inter-hospital variations in LOS for AMI hospitalization in China, with only a small amount of heterogeneity in patient characteristics and risk profiles. This has been similarly observed in Western countries, where healthcare practice is considered more standardized [42–45]. Moreover, the characteristics indicating a hospital’s capacity for AMI treatment were not found to be associated with hospital RS-LOS either, which was also observed in another study [44]. This suggests that a hospital’s organizational or operational strategies – or conventional institutional practices – may play a substantial role in determining how long a patient with AMI stays in the hospital. Of note, the exclusion rate based on the criteria in Figure 1 was similar in different geographic regions or hospitals with relatively long vs. short RS-LOS, so differential selection of patients would not explain the variations by region or hospital.

In the era of medical resource constraint in China, it is important to explore opportunities where greater efficiency could be recognized. Clinical pathways may be one solution, which are management plans that standardize the sequence and timing of the care process to achieve optimal treatment effects for patients and efficiency for hospitals. They are designed based on clinical guidelines and the best evidence from health services research [5]. Since 2009, the Chinese National Health and Family Planning Commission has designed clinical pathways for 331 diseases under 22 disciplines, including AMI, and started implementing them on a trial basis in public hospitals [35]. The goal is to improve healthcare quality and efficiency, and potentially reduce variations in care. Previous research in China and other countries has shown that clinical pathways could improve treatment outcomes and, in particular, reduce LOS for a variety of major health conditions [5, 46–48]. Therefore, it might be helpful to strengthen and expand the implementation of clinical pathways to more hospitals outside of a trial basis in China to improve LOS for patients with AMI. On a larger scale, clinical pathways management is part of an attempt to implement DRG-based payment systems in China to reduce medical costs [49], with the expectation, in part, of reducing unnecessarily extended length of stay, as was seen in countries like the U.S. and Germany after the introduction of these systems [50, 51]. In China, DRG payment has been established on a trial basis in selected hospitals and diseases since 2004 [52]. However, over the years, it has made little progress and still has many barriers in terms of implementation in general practice [52].

Although prolonged LOS is associated with increased consumption of healthcare resources that may or may not translate into better health outcomes, a recent large study conducted among U.S. Veterans Affairs hospitals suggested that hospitals that tend to discharge patients sooner than expected also have modestly higher readmission rates [20]. Furthermore, a prior study also showed that country-level median LOS was associated with a 14% reduction in the odds of readmission for each additional day in hospital [3]. These highlight that future activities to rationalize hospitalization LOS in China should be designed and implemented appropriately, avoiding improving hospital patient flow at the cost of suboptimal patient outcomes.

Our study has several strengths. First, to our knowledge, this is the first study in China that thoroughly examined LOS for AMI using a nationally representative sample of patients from both secondary and tertiary hospitals. Data from the three study years also enabled us to study the trend in LOS for AMI over the past decade. Second, the China-PEACE study implemented rigorous data quality monitoring with methods commonly used for clinical trials to improve not only the completeness but also the accuracy of data extraction [25]. Most patient characteristics that have been previously suggested to be associated with LOS for AMI are captured in our data and included in the risk standardization model [44, 53–55]. However, we also acknowledge that the quality of medical chart documentation might be uneven, as there is much heterogeneity across hospitals in China and could be influenced by physician practice. Finally, the collaboration with the Chinese government on China-PEACE will lead to easier translation of our findings into policies and programs to reduce hospital LOS for AMI. On the other hand, the study was not without limitations. We did not have information on certain hospital characteristics that may be very relevant to our analyses, such as whether an AMI clinical pathway was established in the hospital in a given year. Also, the cross-sectional nature of the current study prevented us from examining the association between LOS and patient prognoses, such as readmission or post-discharge life quality, on patient or hospital level.

## Conclusions

Although decreasing in the past decade, hospital LOS for patients with AMI in China is still long relative to most countries in the world. There is much inter-hospital variation in LOS for AMI hospitalizations. In the context of the growing number of AMI hospitalizations in China, it is critical to rationally shorten LOS, reduce variations in practice among hospitals, and ultimately improve the efficiency of Chinese medical resource utilization.

## Authors’ information

Harlan M Krumholz and Lixin Jiang are joint senior authors.

## Electronic supplementary material

Additional file 1: **Contains the model coefficients and the residual plot of the risk-standardization model for LOS.** (DOCX 109 KB)

Additional file 2: **Contains a figure entitled “Risk-standardized Length of Stay across Secondary Hospitals”, which graphically describes the pattern of variation in RS-LOS for patients with AMI in China across the secondary hospitals in our study sample.** (DOCX 39 KB)

Additional file 3: **Contains a figure entitled “Risk-standardized Length of Stay across Tertiary Hospitals”, which graphically describes the pattern of variation in RS-LOS for patients with AMI in China across the tertiary hospitals in our study sample.** (DOCX 39 KB)

## Abbreviations

AMI: Acute myocardial infarction
LOS: Length of stay
RS-LOS: Risk-standardized LOS
PEACE: Patient-centered Evaluative Assessment of Cardiac Events
CABG: Coronary artery bypass graft
PCI: Percutaneous coronary intervention
GEE: Generalized estimating equation
CI: Confidence interval
IQR: Interquartile range
DRG: Diagnosis related group
STEMI: ST-segment elevation myocardial infarction
COPD: Chronic obstructive pulmonary disease
eGFR: Estimated glomerular filtration rate
CK: Creatine kinase
CK-MB: Creatine kinase MB fraction
ACEI: Angiotensin converting enzyme inhibitor
ARB: Angiotensin receptor blocker.
