# Supplementary material for: National trends in hospital length of stay for acute myocardial infarction in China
Source: BMC Cardiovasc Disord. 2015 Jan 20;15:9. doi: 10.1186/1471-2261-15-9 (PMC4360951; doi:10.1186/1471-2261-15-9)

The length of stay (LOS) was considered of log normal distribution and the risk-standardization model was run using the PROC GLIMMIX in SAS with LOS as the outcome and the ‘link=log’ specification. Therefore, the model coefficients as well as the residuals are on the log scale of LOS and the interpretation of the exponential of each coefficient would be ratio of the LOS comparing a specific characteristic category with its reference category.

The coefficients for the variables included in the risk-standardization model, the exponential of the coefficients, and the p-values are shown in the table below.

| Variable | Coefficient | Exp(coefficient) | p-value |
| --- | --- | --- | --- |
| Intercept | 2.6700 | -- | <0.0001 |
| Year |  |  |  |
| 2001 | 0.2231 | 1.2499 | <0.0001 |
| 2006 | 0.0769 | 1.0799 | <0.0001 |
| 2011 (reference) | -- |  |  |
| Age, years |  |  |  |
| <55 | -0.0538 | 0.9476 | 0.0005 |
| 55-64 | 0.0143 | 1.0144 | 0.2992 |
| 65-74 | 0.0045 | 1.0045 | 0.7145 |
| ≥65 (reference) | -- |  |  |
| Male vs. female | 0.0293 | 1.0297 | 0.0060 |
| Place of residence |  |  |  |
| Urban | 0.0817 | 1.0851 | 0.0012 |
| Rural | 0.0328 | 1.0334 | 0.1797 |
| Unrecorded (reference) | -- |  |  |
| Current smoking | -0.0312 | 0.9693 | 0.0027 |
| Hypertension | 0.0537 | 1.0552 | <0.0001 |
| diabetes | 0.0802 | 1.0835 | <0.0001 |
| History of MI | -0.0264 | 0.9739 | 0.1482 |
| History of PCI | -0.0302 | 0.9703 | 0.3759 |
| History of CABG | 0.0782 | 1.0813 | 0.2480 |
| History of stroke | 0.0068 | 1.0068 | 0.6524 |
| Chronic renal disease | 0.1014 | 1.1067 | 0.0049 |
| History of cancer | -0.0677 | 0.9345 | 0.2528 |
| Symptom onset to admission, hours |  |  |  |
| ≤6 | 0.0605 | 1.0623 | <.0001 |
| 6-12 | 0.0025 | 1.0025 | 0.8746 |
| 12-24 | 0.0264 | 1.0268 | 0.0624 |
| >24 (reference) |  |  |  |
| STEMI | 0.0509 | 1.0522 | 0.0002 |
| Chest pain at admission | 0.0462 | 1.0473 | 0.1537 |
| Cardiogenic shock at admission | 0.0507 | 1.0520 | 0.0289 |
| Cardiac arrest at admission | 0.0875 | 1.0914 | 0.0452 |
| Pneumonia at admission | 0.0328 | 1.0334 | 0.0364 |
| Exacerbated COPD at admission | 0.0265 | 1.0269 | 0.5524 |
| Acute stroke at admission | 0.1303 | 1.1392 | 0.0001 |
| eGFR at admission, ml/(min*1.73m2) |  |  |  |
| >90 | 0.0646 | 1.0668 | 0.0001 |
| 60-90 | 0.0714 | 1.0740 | <.0001 |
| <60 | 0.0099 | 1.0099 | 0.7962 |
| Unknown (reference) | -- |  |  |
| SBP >180 mmHg or DBP >110 mmHg at admission | -0.0359 | 0.9647 | 0.0791 |
| Heart rate >100 beats/min at admission | 0.0250 | 1.0253 | 0.0811 |

MI: myocardial infarction; PCI: percutaneous coronary intervention; CABG: coronary artery bypass graft; STEMI: ST-segment elevation myocardial infarction; COPD: chronic obstructive pulmonary disease; eGFR: estimated glomerular filtration rate; SBP: systolic blood pressure; DBP: diastolic blood pressure.

The residual plot of the risk-standardization model is as follows, which shows that the residuals are approximately evenly scattered above and below 0 without a particular pattern.


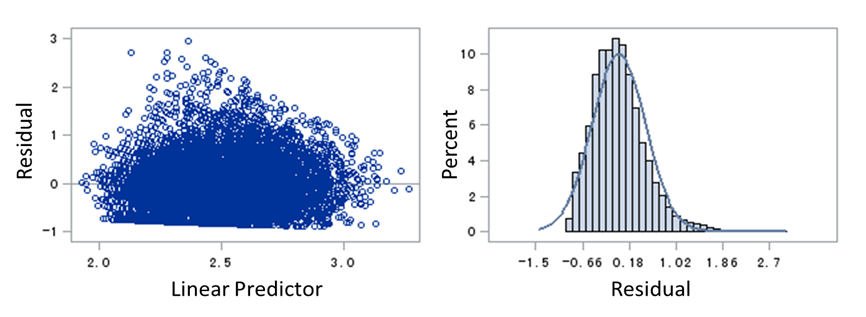

Supplement: Supplementary file 1 — Additional file 1: Contains the model coefficients and the residual plot of the risk-standardization model for LOS. (DOCX 109 KB) [file 12872_2014_849_MOESM1_ESM.docx]
