# Supplementary figures and images for: National trends in hospital length of stay for acute myocardial infarction in China
Source: BMC Cardiovasc Disord. 2015 Jan 20;15:9. doi: 10.1186/1471-2261-15-9 (PMC4360951; doi:10.1186/1471-2261-15-9)

**Figure S1. Risk-standardized Length of Stay across Secondary Hospitals**


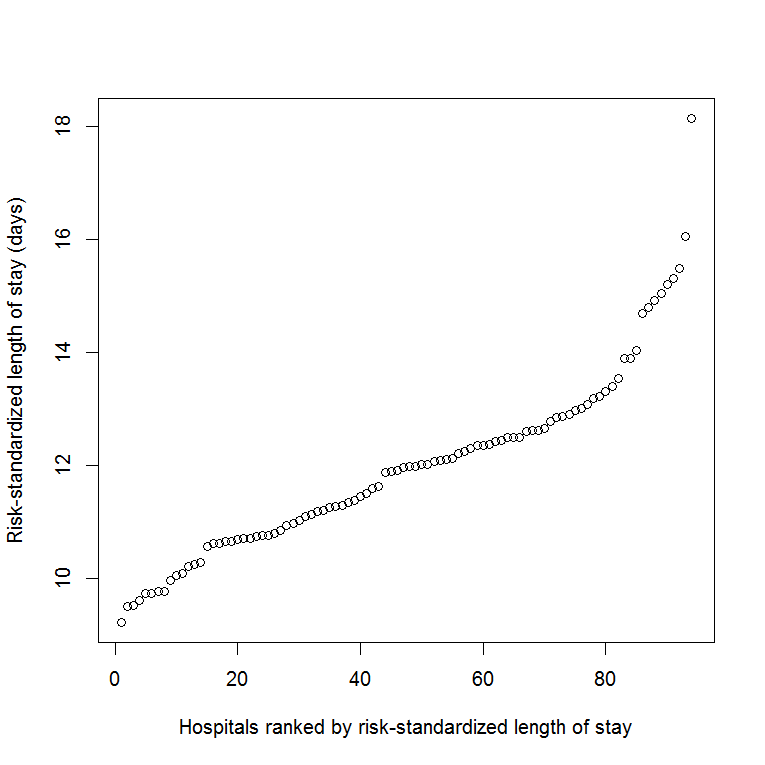

Supplement: Supplementary file 2 — Additional file 2: Contains a figure entitled “Risk-standardized Length of Stay across Secondary Hospitals”, which graphically describes the pattern of variation in RS-LOS for patients with AMI in China across the secondary hospitals in our study sample. (DOCX 39 KB) [file 12872_2014_849_MOESM2_ESM.docx]

**Figure S2. Risk-standardized Length of Stay across Tertiary Hospitals**


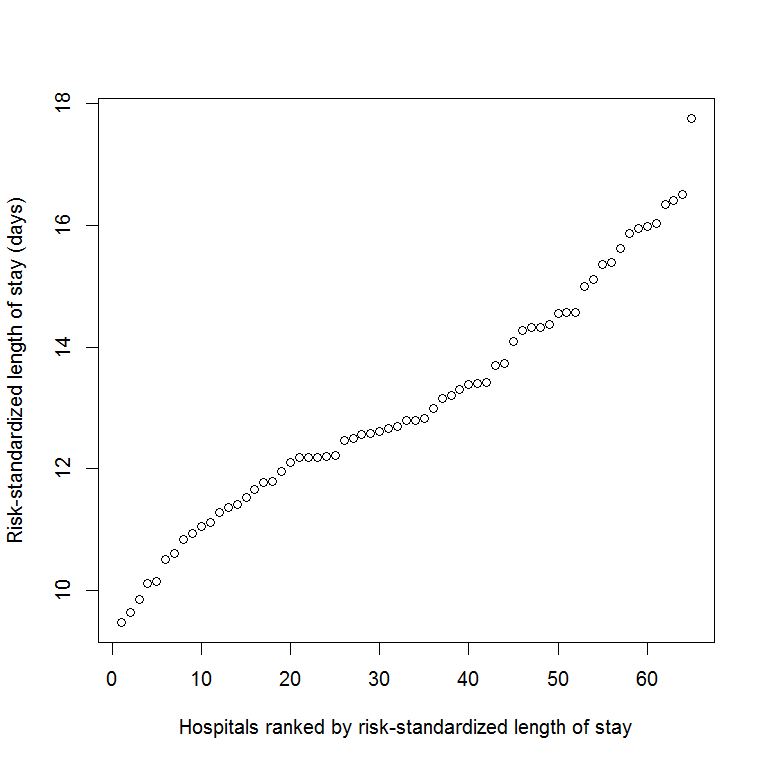

Supplement: Supplementary file 3 — Additional file 3: Contains a figure entitled “Risk-standardized Length of Stay across Tertiary Hospitals”, which graphically describes the pattern of variation in RS-LOS for patients with AMI in China across the tertiary hospitals in our study sample. (DOCX 39 KB) [file 12872_2014_849_MOESM3_ESM.docx]
